# Microdisk array based Weyl semimetal nanofilm terahertz detector

**DOI:** 10.1515/nanoph-2022-0227

**Published:** 2022-07-20

**Authors:** Qi Song, Zhiwen Zhou, Gangyi Zhu, Huawei Liang, Min Zhang, Bingyuan Zhang, Fang Liu, Peiguang Yan

**Affiliations:** College of Physics and Optoelectronic Engineering, Shenzhen University, Shenzhen 518060, China; Shandong Key Laboratory of Optical Communication Science and Technology, School of Physics Science and Information Technology, Liaocheng University, Liaocheng 252059, China; School of Communication and Information Engineering, Nanjing University of Posts and Telecommunications, Nanjing 210003, China; Beijing Key Laboratory of Passive Safety Technology for Nuclear Energy, School of Nuclear Science and Engineering, North China Electric Power University, Beijing 102206, China

**Keywords:** Weyl semimetal, microdisk array, terahertz detector

## Abstract

High-performance terahertz wave detectors at room temperature are still urgently required for a wide range of applications. The available technologies, however, are plagued by low sensitivity, narrow spectral bandwidth, complicated structure, and high noise equivalent power (NEP). Here, we have demonstrated a Weyl semimetal surface plasmon-enhanced high-performance terahertz wave detectors which are based on microdisk array deposited WTe_2_ nanofilm epitaxially grown on GaN substrate for room temperature operation. With the microdisk array combined the WTe_2_ layer, strong terahertz wave surface plasmon polaritons can be generated at the WTe_2_–air interfaces, which results in significant improvement in detecting performance. For the 40 μm diameter microdisk array, a detectivity (*D* ^*^) of 5.52 × 10^12^ cm Hz^1/2^ pW^−1^ at 0.1 THz is achieved at room temperature. In addition, the responsivity (*R*_A_) of 8.78 A W^−1^ is also obtained. Such high-performance millimeter and terahertz wave photodetectors are useful for wide applications such as high capacity communications, walk-through security, biological diagnosis, spectroscopy, and remote sensing.

## 1 Introduction

Photodetectors are widely used in modern military and civil fields. Limited by the bandgap of photoelectric materials, the photoelectric detection ability of traditional semiconductor materials can only cover a certain wavelength region [1–4]. There are many technical bottlenecks in some special frequency bands, especially in the THz bands (0.1–10 THz). Since the first graphene photodetector in 2009 [5], researchers have carried out extensive research on semimetal photodetectors in the past decade [6–8]. The semimetal has many advantages in key properties, such as wideband or even could be used in full band detection, especially in the mid and far-infrared detection at room temperature [9–12]. The response time can also be reduced to a picosecond, which is suitable for terahertz ultrafast optical communication and optical interconnection [13–16]. On the other hand, the ordinary semimetal has no bandgap, and the large dark current is not conducive to the application of large bias voltage, which restricts the improvement of its responsivity [17, 18]. In the past, although various special structural designs can improve the responsivity of the detector, they cannot take into account both the wideband response and the ultrafast response speed, thus losing the unique advantages of the semi-metallic detector [2, 4]. It is a very important task to improve the sensitivity considering the wide spectrum and ultrafast response of the semimetal detector at the same time. Compared with two-dimensional material like graphene, the Weyl semimetal photodetector maintains a wideband response from visible light to mid-infrared and a picosecond ultrafast response [19, 20]. In addition, due to the stronger interaction with light, the responsivity is also improved by an order of magnitude. In principle, there is a divergent berry curvature near the Weyl point in the Weyl semimetal, which makes the displacement current response related to the berry field significantly enhanced near the Weyl point [19, 21]. The lower the energy such as THz frequencies, the closer the transition will be at the Weyl point, and the more obvious the enhancement effect could be obtained. Therefore, Weyl semimetal is a kind of suitable material for terahertz detector. To improve the capability of devices in modulating interactions between light and matter, subwavelength structures are a lot to be used [22, 23]. In addition, combined with the lower plasma frequency (such as graphene) property of Weyl-semimetal and the subwavelength structures [2, 4], detection of terahertz waves was more suitable. Structures adoption for plasmonic materials in longer wavelength is of potential to facilitate the photoelectric conversion capability. The type-II Weyl material WTe_2_, with bulk Weyl nodes connected by Fermi-arc surface states, shows an in-plane spin conductivities of 7.36 × 10^3^ (ℏ/2e) (Ωm)^−1^, have been revealed as a new contender for photodetector innovations [24, 25].

Here, we have designed a Weyl semimetal surface plasmon-enhanced high-performance terahertz wave detector which is based on a microdisk array deposited WTe_2_ nanofilm deposited on GaN substrate for room temperature operation. With a microdisk array combined with the WTe_2_ layer, strong terahertz wave surface plasmon polaritons can be generated at the WTe_2_–air interfaces, which results in significant improvement in detecting performance [22, 23]. The microdisk array figures of merits *R*_A_ (8.78 AW^−1^), noise equivalent power (NEP) (0.74 pW Hz^−1/2^), and *D* ^*^ (0.41 × 10^12^ cm Hz^1/2^ W^−1^) show good performances than the commercial detectors or even low-temperature detector (Golay cell *R*_v_ = 1 × 10^5^ V W^−1^, NEP = 140 pW Hz^−1/2^, low-temperature Bolometer NEP = 0.25 pW Hz^−1/2^, Schottky diodes NEP = 15.2 pW Hz^−1/2^ and thermal-type detector *D* ^*^ = 1.9 × 10^10^ cm Hz^1/2^ W^−1^). The effective detection area of the microdisk device is 6.5 × 1.5 mm, which makes our device more suitable for practical applications. Combined with the stability and simple manufacturing method and low cost, our detector can be employed as an alternate for commercial detectors in the terahertz communication, sensing, and imaging field.

## 2 Characterization and preparation of Microdisk array based Weyl semimetal nanofilm and experimental setup

The devices were fabricated by two main processes. The GaN microdisk array was fabricated by photolithography based on a GaN-on-silicon wafer. The WTe_2_ and electrode (Au) layers were deposited on the GaN microdisk array by the magnetron sputtering deposition method. Detailed processing processes are as follows. Firstly, the photoresist was deposited on the substrate accompanied by a spatial notch (length of 2 μm) marked. Secondly, a 200 nm nickel film was also deposited on the substrate employed as a hard mask. Thirdly, the GaN layer was etched on the silicon layer. The etching rate of 5 nm/s was used. Gases and volume flux are argon, chlorine, and boron trichloride; 30, 20, and 20 sccm, respectively. The power, pressure, and etching time are 500 W, 60 mTorr, and 7 min, respectively. Fourthly, using dilute nitric acid to remove the nickel film. Finally, a gap under the GaN layer was made by the HNF solution isotropic wet etching process. In this paper, we use the microdisks array device covered with WTe_2_ and single WTe_2_ layer device as the reference.

**Figure 1:**
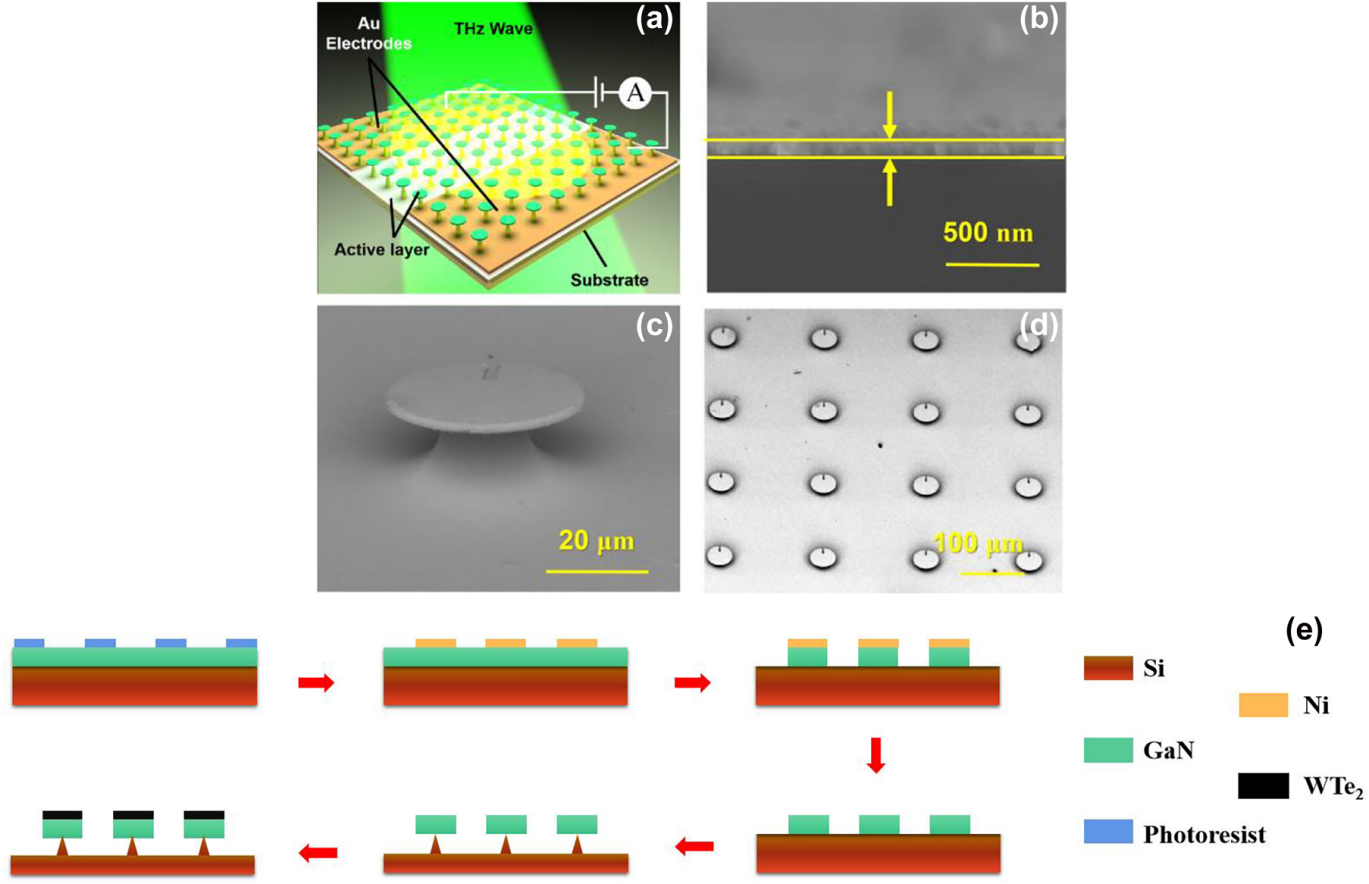
Device characterization and preparation processes. (a) Schematic diagram of microdisks array covered with WTe_2_. (b) The thickness of the WTe_2_ film. (c) and (d) SEM pictures at oblique view. (e) The device processing setup.

**Figure 2:**
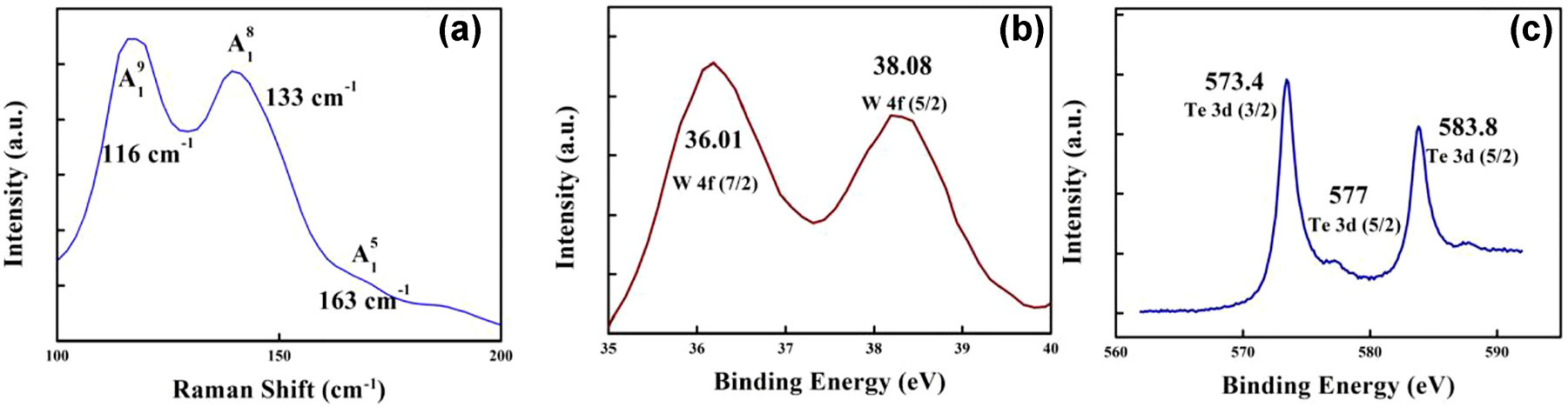
WTe_2_ device characterization. (a) Raman spectra. (b) and (c) XPS spectra.

In addition, the tapered pillars are used to support microdisks. The WTe_2_ layer and Au electrode were deposited by the following steps. The vacuum degree was reduced to 9 × 10^−4^ Pa and injects argon into the cavity. The WTe_2_ target is coated by RF drive, and the Au target is coated by DC drives. For the WTe_2_ layer, the argon flow rate, power, and duration time were 50 SCCM, 100 W, and 180 s. For the Au electrode layer, the argon flow rate, current, and duration time of 15 SCCM, 0.2 A, and 90 s were used. In order to improve the stability of the device, we prepared a SiO_2_ film with the thickness of 70 nm in the detection region by magnetron sputtering deposition. Figure 1 shows the device design. The thickness of the WTe_2_ film is 74 nm. The SEM pictures had shown that the diameter of the microdisk was 40 μm with the transverse spacing and longitudinal spacing of 150 and 100 μm. A CW THz emitter (*TeraSense IMPATT diodes*) with the frequency of 0.1 THz was employed as the THz source. A high-precision picoammeter (*Keithley 6485*) was used to record the light and dark current. A linear DC power supply (*Rigol DP832A*) was used as the power supply. The detector was set on a tungsten steel probe platform and the THz emitter was vertically incident (angle of incidence normal to the microdisk face) on the detector. Figure 2 shows the device-based WTe_2_ film characterization. Raman spectroscopy was used to characterize the WTe_2_ deposited device under a 532 nm laser. The Raman peaks located at around 116, 133, and 163 cm^−1^ (Figure 2a) contribute to the vibration modes of $A_{1}^{9}A_{1}^{8}$ , and $A_{1}^{5}$ , respectively (consistent with Ref. [26]). X-ray photoelectronic spectroscopy (XPS) was employed to demonstrate the surface chemical states of WTe_2_ film. XPS spectra are shown in Figure 2b and c. Figure 2b indicated two peaks at 36.06 and 38.1 eV for W 4*f* (7/2) (metal) and W 4*f* (5/2), respectively. Figure 2c indicated three distinct peaks at 573.4, 577 and 583.8 eV for Te 3*d* (3/2) (metal), Te 3*d* (5/2) (TeO_2_) and Te 3*d* (5/2) (metal), respectively [27]. The reference device and microdisk device have the same size, electrode spacing, material thickness, and WTe_2_ coating parameters.

## 3 Results and discussion

**Figure 3:**
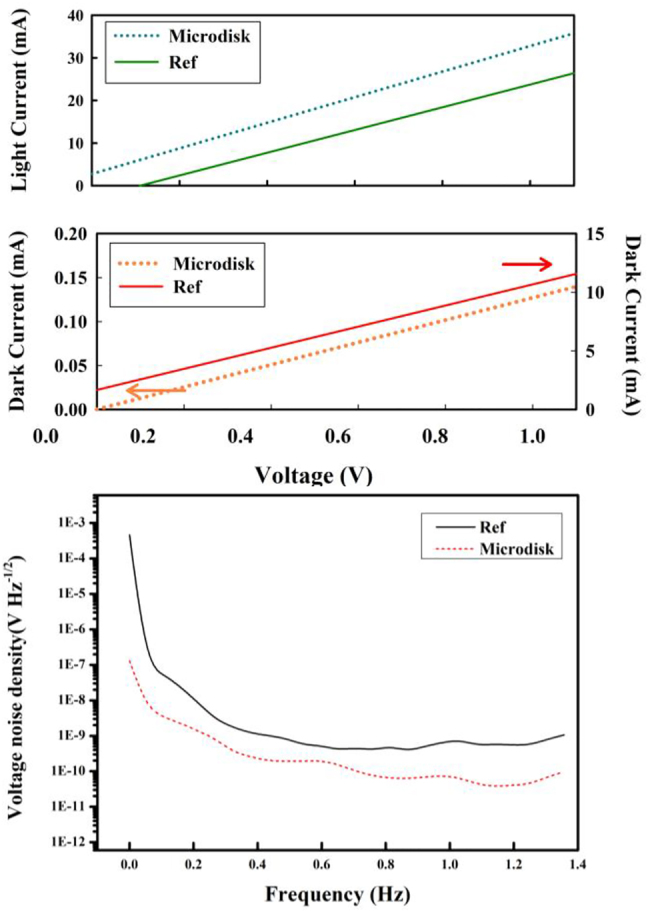
Light current, dark current with the applied voltage and noise characterization with the frequency at room temperature.

Figure 3 shows the light current, dark current, and noise characterization. In the dark Ampere–Volt date, a good ohmic character of these devices could be observed. The microdisks array device and single WTe_2_ layer device (as the reference) exhibit a remarkable difference in the dark current level, indicating strong effects of the microdisks array. To further identify the noise, the noise current by using Fourier transform (FFT) of time-dominated (20 s) dark current at the bias voltage of 1 V are demonstrated. As shown in Figure 3, the microdisk array device and reference device have 1/f noise voltages of 1.2 and 14.1 nV Hz^−1/2^, respectively. The device noise also include the thermal Johnson–Nyquist noise and dark current shot noise [4]. After 1 Hz, the white noise will dominate the total noise. The THz detector figures of merit photoresponsivity (*R*_A_), noise equivalent power (NEP), and detectivity (*D* ^*^) were used to evaluate the photoelectrical conversion capability of the detector. These figures of merits of *R*_A_, NEP, and *D* ^*^ could be expressed as [2, 4]:

(1) $$R_{A}=I/P$$

(2) $$NEP=V_{n}/R_{A}$$

(3) $$D^{*}=\sqrt{S}/NEP.$$

**Figure 4:**
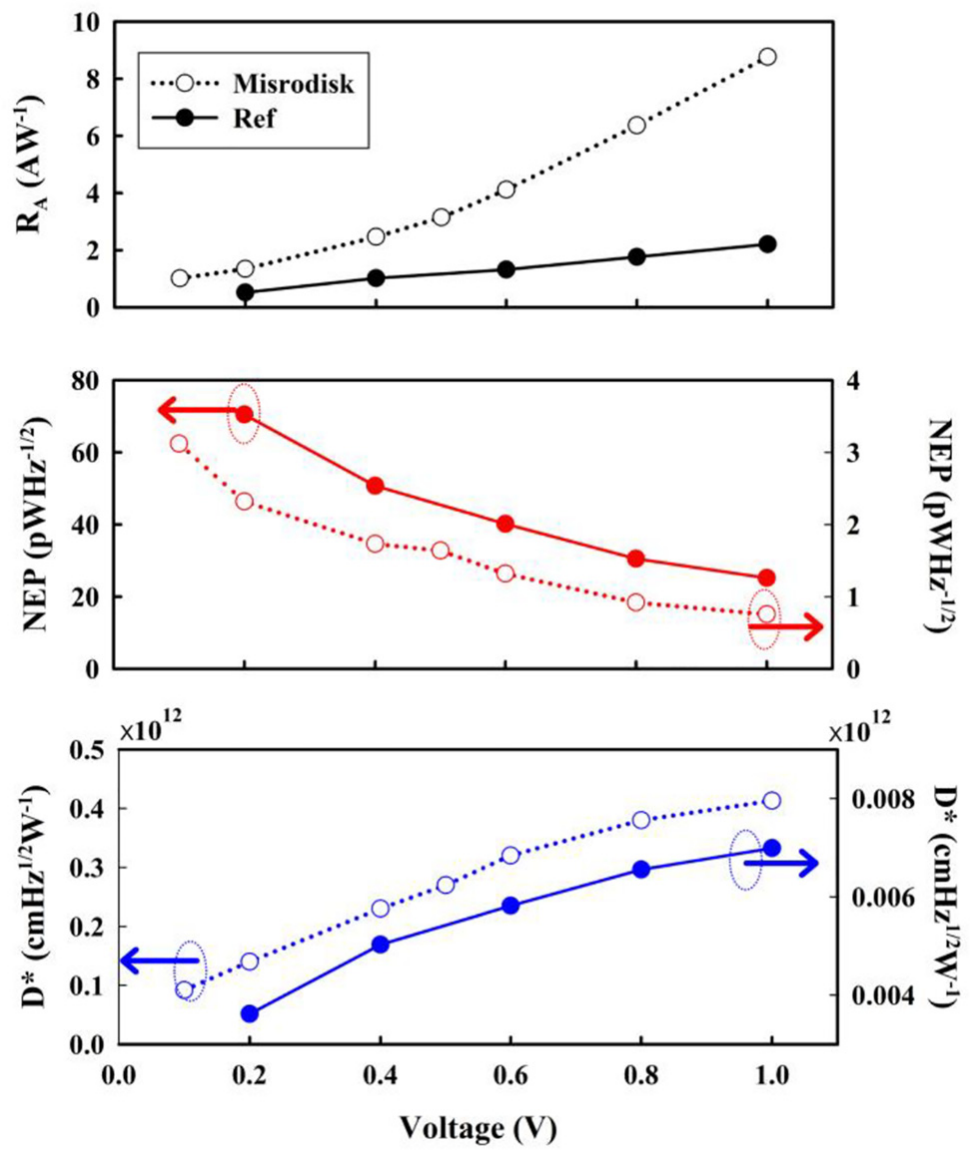
*R*_A_, NEP, and *D* ^*^ of the devices at different voltage bias at room temperature.

*I* is the photocurrent of the device. *P* is the incident THz power, *S* is the effective detection area of the detector. And *V*_n_ is the noise voltage. Figure 4 shows the performance versus bias. *R*_A_, NEP, and *D* ^*^ of the devices at different voltage biases for room temperature operation at 0.1 THz. The microdisk array represents a strong performance enhancement of almost 4 times on *R*_A_ and 30 times on NEP compared to the reference sample. Due to the nonequilibrium electrons proportional to the drift velocity. With the voltage of 1 V, *R*_A_ of the microdisk array device and reference sample are 8.78 A W^−1^ and 2.21 A W^−1^, respectively. The corresponding *R*_v_ is 6.14 × 10^5^ V W^−1^ of the microdisk array which is higher than that of a Golay cell (1 × 10^5^ V W^−1^). In addition, the *R*_v_ is also 2 times more than the best results of microstructure devices [4]. It is an excellent room temperature detector. In addition, due to an increased noise level, NEP and *D* ^*^ show enhancement with the increasing voltage and tend to saturate with the voltage close to 1 V. With the voltage of 1 V, NEP of the microdisk array device and reference sample are 0.74 pW Hz^−1/2^ and 25.2 pW Hz^−1/2^, respectively. The low NEP is just 1/189 than a commercial Golay cell (140 pW Hz^−1/2^) [2], only 3 times more than a commercial Bolometer (0.25 pW Hz^−1/2^) [2] at low temperature and 1/21 than a commercial Schottky diode (15.2 pW Hz^−1/2^) [2]. The *D* ^*^ reaches 0.41 × 10^12^ Hz^1/2^ W^−1^ which is 20 times more than the commercial thermal-type detector (1.9 × 10^10^ cm Hz^1/2^ W^−1^) [4]. The microdisk array figures of merits *R*_A_, NEP, and *D* ^*^ show better performances than the commercial detectors or even low-temperature detectors. Combined with the stability and simple manufacturing method and low cost, our detector can be employed as an alternate for commercial detectors in the terahertz communication, sensing, and imaging field.

**Figure 5:**
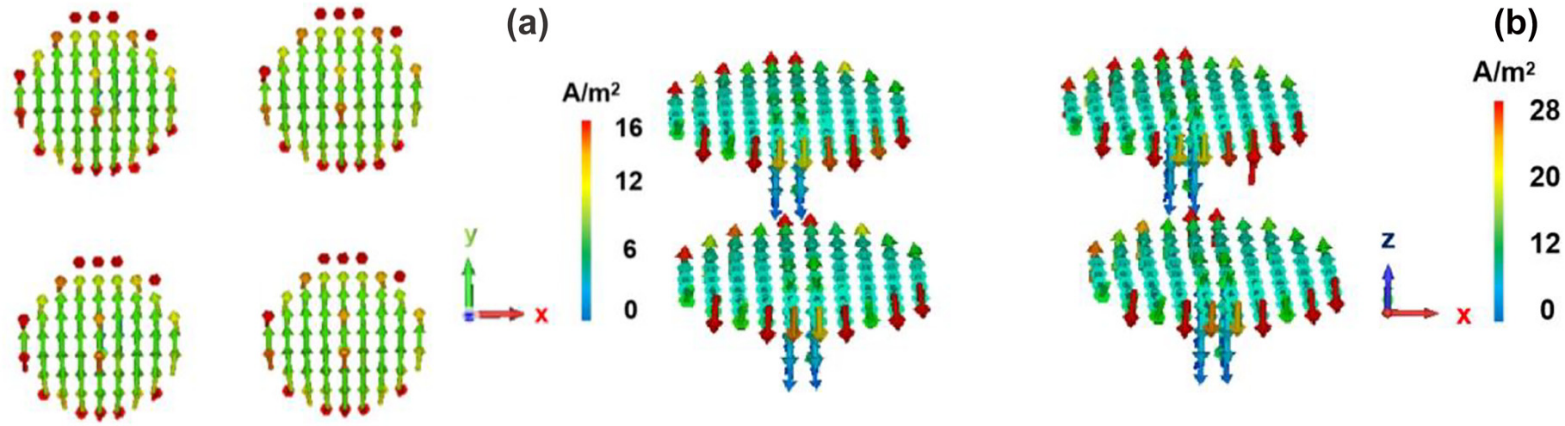
Current density direction on the surface of microdisk array. (a) Top and (b) front views.

If there are some fine structures on the interface between metal and medium, localized surface plasmon (LSP) localized on the surface of the fine structure will be generated. LSP can bind the energy of the electromagnetic field to a very small scale, which mainly exists on small particles with a closed surface. The frequency of LSP is related not only to the material of metal and medium but also to the shape and scale of microstructure at the interface of metal and medium. Microdisk is designed with the height of 20 μm and diameter of 40 μm. For the incident terahertz wave (0.1 THz), in the subwavelength range, the surface plasmon can be generated [28–32]. The incident terahertz photons interact with the carriers in the Weyl semimetal layer and form surface plasmons on the surface of the detector array. The direction perpendicular to the device plane is defined as the Z direction. As can be seen from Figure 5a, the generated photocurrent direction is in the XY plane, the surface plasmon generated by the sub-wavelength microdisk structure forms a standing wave in the disk, and the current density is concentrated at the edge of each microdisk. The carriers diffuse to the edge of the disk and concentrate on the edge, and then diffuse downward along the pillar. In addition, the microdisk pillar provides a path for the photocurrent on the disk surface, as shown in Figure 5b. Therefore, the microdisk array with the Weyl semimetal layer leads to the high response of the whole detector.

**Figure 6:**
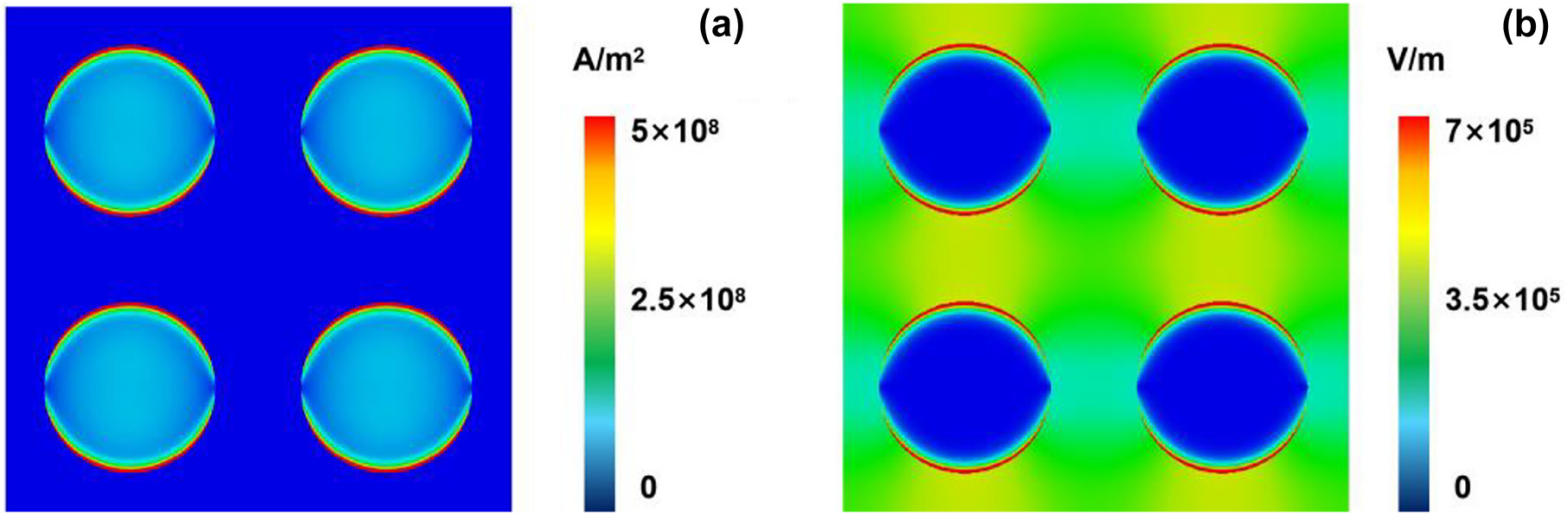
Simulation results. (a) Surface current distribution of the devices, (b) electric field distribution.

**Figure 7:**
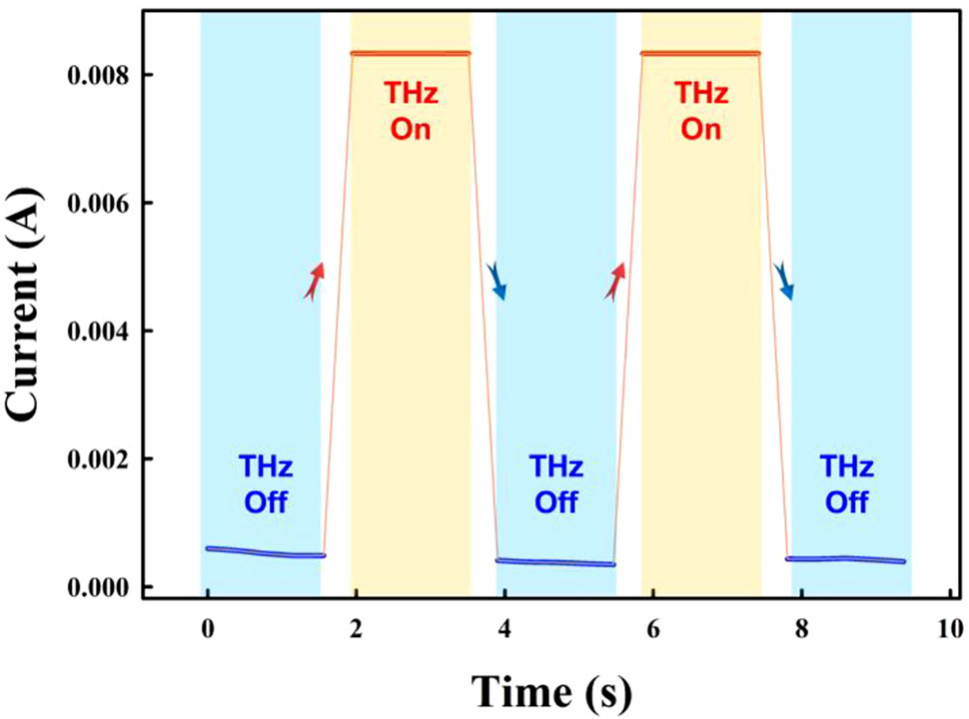
The response time of the device.

Figure 6a and b shows the simulation of surface current and electric field at 0.1 THz. The electric field is more concentrated on the edge of the microdisk and around the microdisk on the substrate. According to the analysis of the surface current intensity, the microdisk array with WTe_2_ deposited, which combined the structure and Weyl-semimetal properties, can be employed for 0.1 THz frequency detection. In addition, the response time of the microdisk array device is shown in Figure 7. The response time of 200 ms 0.1 THz testing by the picoammeter (Keithley 6485) was obtained. Limited by the current test sampling frequency of the instruments, which lead to the longer electrical signal response time.

Table 1 compared with the existing detectors, the detector proposed in this work has certain advantages in NEP and detection sensitivity. The stability of our devices is well, we tested the device after 2 weeks, and the light and dark currents did not perceptible changed.

**Table 1:** THz detectors based on different materials in recent five years.

Materials	Types	NEP pW/	*R*_A_/*R*_v_	Frequency	*D* ^*^ cm^2^	Ref	Response
		Hz^1/2^		THz	Hz^−1/2^ W^−1^		time
Graphene	Nonlinear Hall effect	34	764 V W^−1^	0.45		[33]	**–**
Graphene transistor	Thermoelectric	100	280 V W^−1^	0.12		[34]	4 μs
	terahertz detector						
Graphene	P–N junction	80	105 V W^−1^	1.8–4.2		[35]	30 ns
Bi_2_Se_3_	Electromagnetic	0.36	475 A W^−1^	0.3	2.17 × 10^11^	[36]	60 ms
	induction well						
AlGaN/GaN	Bow-tie Tera-field	25	–	0.504		[37]	**–**
				effect transistor (FET)								
31	0.6											
Selenium doped black	Thermoelectric terahertz	7000	3 V W^−1^	3.4		[38]	**–**
		
phosphorus	detector						
Self-assembled Sn-nanothreads	GaAs FET	6.5	1.3 A W^−1^	0.14	6.7 × 10^8^	[39]	
							
Bi_2_Te_3_	Electromagnetic induction well	1.75	10 V W^−1^	0.332		[40]	8 μs
PtTe_2_	Van der Waals heterojunction	10	1400 V W^−1^	0.12		[41]	9 μs
PdTe_2_	Phototransistor	57	1.3 × 10^−8^ V W^−1^	0.3		[42]	ms–μs
Black phosphorus	Photothermoelectric effect	138	297 V W^−1^	0.29		[43]	0.8 ms
Carbon nanotubes	Flexible FET	12,000	0.22 V W^−1^	3.1		[44]	
				3.8			
Graphene	Nanomesh FET	–	2.5 A W^−1^	3		[45]	
InSb	Plasma nano-groove array	0.02	266,000 V W^−1^	0.171	2.7 × 10^12^	[4]	780 ns
WTe_2_	Plasma microdisk array	0.74	614,000 V** **W^−1·^	0.1	0.41 × 10^12^	This work	200 ms

## 4 Conclusions

In conclusion, we have investigated high-performance terahertz wave detectors which are based on microdisk array deposited WTe_2_ nanofilm deposited on GaN microdisk array substrate for room temperature operation. With a microdisk array combined with the WTe_2_ layer, strong terahertz wave surface plasmon polaritons can be generated at the WTe_2_–air interfaces, which results in significant improvement in detecting performance. For the 40 μm diameter microdisk array, a detectivity (*D* ^*^) of 0.41 × 10^12^ cm Hz^1/2^ W^−1^ at 0.1 THz is achieved at room temperature. In addition, the NEP and *R*_A_ of 0.74 pW Hz^1/2^ and 8.78 A W^−1^ are also obtained. The effective detection area of the microdisk device is 6.5 × 1.5 mm. Therefore, novel detectors presented in this letter have the potential to facilitate Weyl-semimetal-based nanostructures for high-performance and multifunctional long-wavelength optoelectrical devices.

## Supplementary Material

Supplementary Material Details
