# Supplementary material for: Microdisk array based Weyl semimetal nanofilm terahertz detector
Source: Nanophotonics. 2022 Jul 20;11(16):3595–602. doi: 10.1515/nanoph-2022-0227 (PMC11501346; doi:10.1515/nanoph-2022-0227)
Supplement: Supplementary file 1 — Supplementary Material Details [file j_nanoph-2022-0227_suppl.docx]

Supplementary Materials

**Experimental setup**

The detection area was determined using a terahertz camera that measured the size of the terahertz spot within the same distance from the source to the detector. The terahertz camera, type ophir spiricon PY-III-C-A, was employed to measure the spot area S. For the reliability of the experimental data, the total power (*W*) obtained with a terahertz power meter (ELVA-1 DPM R-1612) in direct contact with the emitting end of the IMPATT diode. The spot area is *S* and the detector area is *s*, so the incident power to the terahertz detector is *W/S×s*.The WTe_2_ layer is covered on the device surface contains the microdisk and the substrate. The Au layer is partly covered the microdisk array in order to well contact with the device and probes.


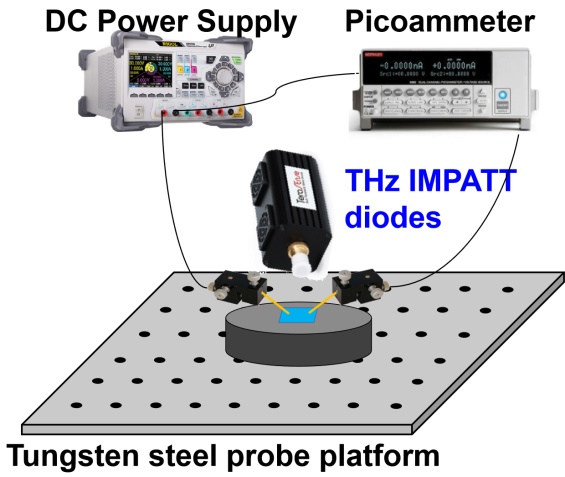


Fig.S1.Schematic diagram of the experimental setup

We use a light-dark current comparison method based on light current excitation to characterize the detection capability of the device (including I-V curve, total noise v_n_, response R_V_, NEP, D^*^, etc.). The detection setup is shown in Fig.S1. The terahertz detector is placed in the center of the probe platform, and two tungsten probes (needle tip of 1 μm) are used to contact the metal electrodes of the device and are connected in series with the DC power supply and the picoammeter. The terahertz avalanche diodes are used as terahertz sources (TeraSense IMPATT [diodes@0.1](mailto:diodes@0.1) THz), and the I-V curves recorded by the picoammeter (keithley 6485). The schematic diagram of the experimental setup is shown in Fig.S1. In addition, We have tested by positive and negative connections, and the experimental results are consistent.

**Reference device**


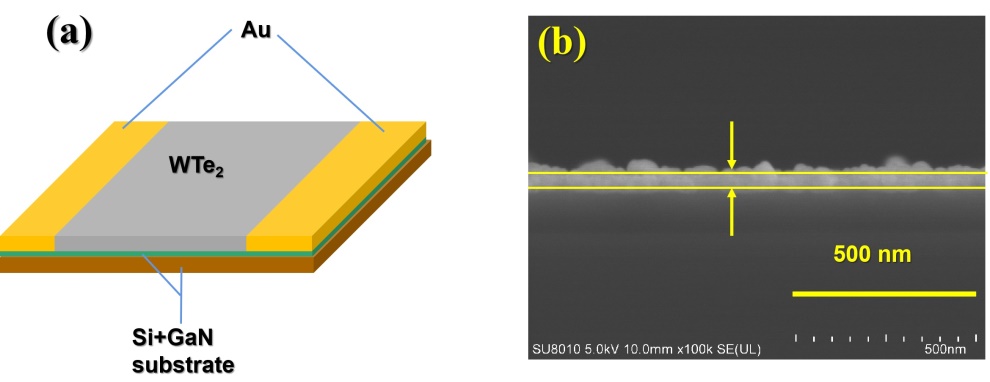


Fig.S2. Reference device diagram and cross section SEM of the WTe_2_ film.

Reference device diagram and cross section SEM of the WTe_2_ film were shown in Fig.S2. The WTe_2_ layer and Au electrode were deposited by the following steps. The vacuum degree was reduced to 9 × 10^-4^ Pa and injects argon into the cavity. The WTe_2_ target is coated by RF drive, and the Au target is coated by DC drives. For the WTe_2_ layer, the argon flow rate, power, and duration time were 50 SCCM, 100 W, and 180 secs. For the Au electrode layer, the argon flow rate, current, and duration time of 15 SCCM, 0.2 A, and 90 secs were used. The thickness of the WTe_2_ film is 71 nm(Roughly the same as the device) and the substrate is the same as the microdisk array.
